# Effects of Lead on the Morphology and Structure of the Nucleolus in the Root Tip Meristematic Cells of *Allium cepa* L.

**DOI:** 10.3390/ijms150813406

**Published:** 2014-07-31

**Authors:** Ze Jiang, Huaning Zhang, Rong Qin, Jinhua Zou, Junran Wang, Qiuyue Shi, Wusheng Jiang, Donghua Liu

**Affiliations:** 1 Tianjin Key Laboratory of Animal and Plant Resistance, College of Life Sciences, Tianjin Normal University, Tianjin 300387, China; E-Mails: xiaoxiaojiangze@126.com (Z.J.); skyzjh@mail.tjnu.edu.cn (J.Z.); wang_junran@126.com (J.W.); qys19891371@126.com (Q.S.); jiangwusheng@mail.tjnu.edu.cn (W.J.); 2 Institute of Genetics and Physiological, Hebei Academy of Agriculture and Forestry Sciences, Shijiazhuang 050051, China; E-Mail: 13821201341@163.com; 3 School of Life Science, South China Normal University, Guangzhou 510631, China; E-Mail: qinrong19870325@163.com

**Keywords:** *Allium cepa* var. *agrogarum* L., lead (Pb), nucleolus, nucleophosmin, nucleolin, fibrillarin

## Abstract

To study the toxic mechanisms of lead (Pb) in plants, the effects of Pb on the morphology and structure of the nucleolus in root tip meristematic cells of *Allium cepa* var. *agrogarum* L. were investigated. Fluorescence labeling, silver-stained indirect immunofluorescent microscopy and western blotting were used. Fluorescence labeling showed that Pb ions were localized in the meristematic cells and the uptake and accumulation of Pb increased with treatment time. At low concentrations of Pb (1–10 μM) there were persistent nucleoli in some cells during mitosis, and at high concentration (100 μM) many of the nucleolar organizing regions were localized on sticky chromosomes in metaphase and anaphase cells. Pb induced the release of particles containing argyrophilic proteins to be released from the nucleus into the cytoplasm. These proteins contained nucleophosmin and nucleolin. Pb also caused the extrusion of fibrillarin from the nucleus into the cytoplasm. Western blotting demonstrated the increased expression of these three major nucleolar proteins under Pb stress.

## 1. Introduction

Lead (Pb) is present in the environment in many forms. It is the most abundant heavy metal contaminant, and is released through a variety of anthropogenic activities [1]. Elevated levels of Pb in soil result from the use of Pb-based paints, shotgun pellets made of Pb, Pb arsenate pesticides, coal burning, gasoline, explosives, Pb batteries and from the disposal of municipal sewage sludge enriched in Pb [2,3]. Considerable attention has been paid to the problems of Pb pollution associated with the development of modern industry and agriculture. Many studies on Pb toxicity in plants indicate that it is linked with disturbance of mitosis [4,5,6,7], toxicity in nucleoli [5,8,9], induction of leaf chlorosis [10,11], inhibition of root and shoot growth [1,12], reduction in photosynthesis [13] and DNA synthesis [14], and alterations in enzymatic activities [13,15].

The nucleolus is a nuclear structure, responsible for ribosome biogenesis and transcription [16]. It can be selectively stained by silver as it contains a set of acidic, nonhistone proteins. Nucleophosmin, fibrillarin and nucleolin are abundant and multifunctional nuclear proteins (NPs) that participate in ribosomal RNA (rRNA) processing and ribosome biogenesis [17,18].

It has been reported that nucleolar material, detected by silver staining and described as “silved-stained particulate material”, is extruded into the cytoplasm in the root tip cells of plants exposed to aluminum (Al) [19], cadmium (Cd) [20] and Pb [5,9]. New evidence obtained by electron microscopy [9] confirmed that silver-stained particles are released from the nucleolus into the cytoplasm. However, limited information is available about the nature of these silver-stained particles and the effects of Pb on nucleolus and NPs, especially what types of NPs are scattered in the nuclei or extruded into the cytoplasm under Pb stress.

*Allium cepa* (2*n* = 16) is not only an agriculturally important plant but is also used as a model [21]. In order to further understand and confirm the cytological effects of Pb on the nucleolus and NPs, the toxic effects of Pb on nucleoli in the root tip cells of *A. cepa* were investigated by silver staining methods. In addition, alteration in the cellular localization and expression of the three major NPs: nucleophosmin, fibrillarin and nucleolin, were examined using indirect immunofluorescence and western blotting.

## 2. Results and Discussion

### 2.1. Localization of Pb in the Root Tip Meristematic Cells

**Figure 1:**
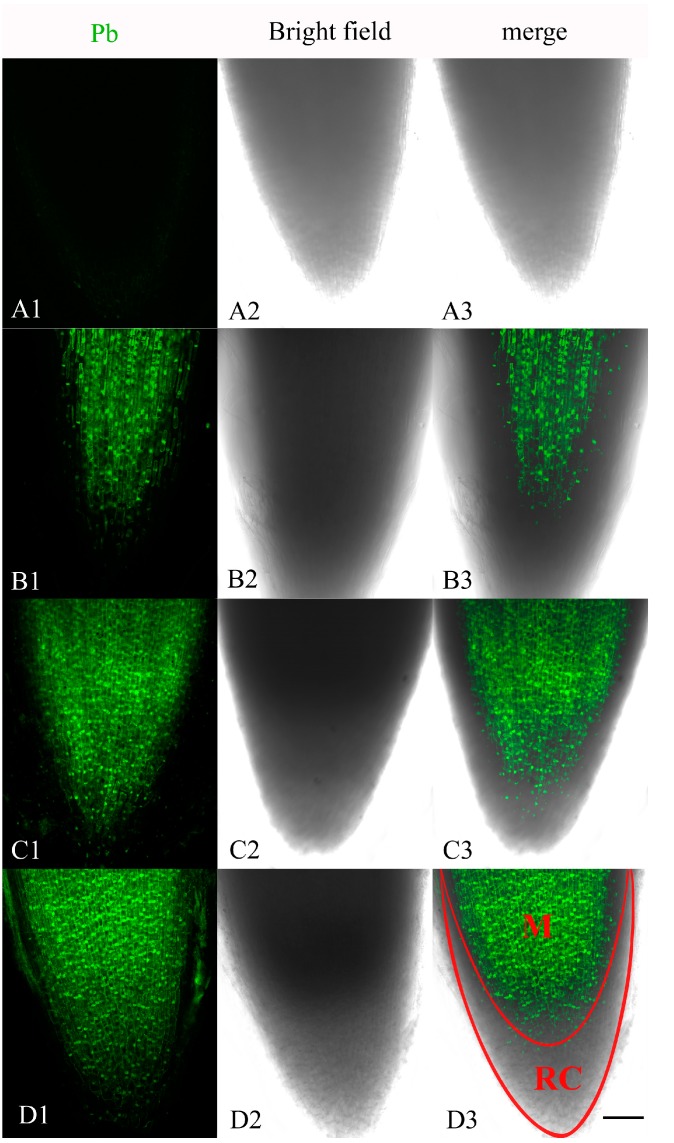
Confocal laser scanning microscope micrographs from root tip meristematic cells of *A. cepa* exposed to Pb(NO_3_)_2_ for 4, 24 and 48 h, using Leadmium™ Green AM dye (Invitrogen). (**A1**,**B1**,**C1**,**D1**) Pb detection; (**A2**,**B2**,**C2**,**D2**) Bright field image; (**A3**,**B3**,**C3**,**D3**) Merged image of “Pb detection” and “bright field image”. All images are taken at ×10 magnification, and green fluorescence represents the binding of the dye to Pb. One of six typical examples is selected for each treatment. Scale bars = 100 μm. M = meristematic zone. RC = root cap.

The distribution of Pb in the root tip meristematic cells of *A. cepa* was examined after exposure for 4, 24 and 48 h to Pb Leadmiu™ Green AM dye (Invitrogen, Carlsbad, CA, USA). The fluorescent dye showed a clear and bright green fluorescence in the root tip cells of Pb-treated plants, whereas no fluorescence reaction was found in control cells (Figure 1A1–A3). A weak green fluorescence labeling of Pb was mainly distributed in the center of meristematic cells exposed to Pb for 4 h (Figure 1B1–B3). By 24 h treatment, the labeling of the meristematic cells increased (Figure 1C1–C3). The most intense fluorescence in meristematic cells was observed after 48 h of incubation (Figure 1D1–D3). These results indicated that Pb ions were localized in meristematic cells and that the uptake and accumulation of Pb increased with treatment time.

### 2.2. Effects of Pb on the Nucleolar Organization Regions

**Figure 2:**
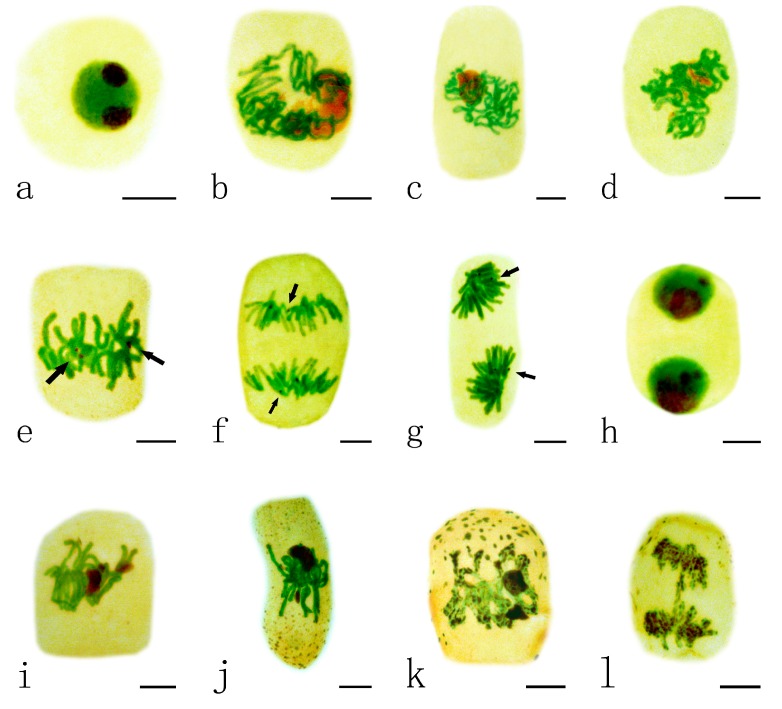
Effects of different concentrations of Pb on nucleolar organizing regions (NORs) in root tip meristematic cells of *A. cepa* during mitosis. (**a**–**h**) Normal mitotic process. (**a**) The interphase cell; (**b**,**c**) Showing decondensed chromatin fibers were around the nucleoli; (**d**) Showing that the nucleoli disappeared in their characteristic structures; (**e**) Showing NORs localized on metaphase chromosomes; (**f**) Showing NORs migrated with the chromosomes to the poles at anaphase; (**g**) Showing nucleoli rebuilt at early telophase and the size increased; (**h**) Showing the two daughter nuclei entered interphase, and mitosis was completed; (**i**–**l**) Mitotic process under Pb stress. (**i**,**j**) Showing persistent nucleoli during metaphase ((**i**) 10 μM Pb, 24 h; (**j**) 1 μM Pb, 48 h);(**k**) Showing sticky chromosomes with Ag-stained NOR particles at metaphase (100 μM Pb, 48 h);and (**l**) Showing sticky chromosomes with Ag-stained NOR particles at anaphase (100 μM Pb, 48 h).Scale bars = 10 μm. Nucleoli and NORs: dark brown; nuclei and chromosomes: green; cytoplasm: yellow. Arrowhead shows NORs.

The nucleolar organizing regions (NORs) were stained as dark-brown particles scattered within the green nuclei. Normally the nucleus of *A. cepa* contains one or two nucleoli and the nucleoli in interphase cells, impregnated with silver, showed strong staining (Figure 2a). With progressing prophase, decondensed chromatin fibers were seen around the nucleoli (Figure 2b–c). During prometaphase, the nucleoli decreased in size, and then their characteristic structures disappeared (Figure 2d). At the time of chromosome formation, in metaphase, the chromosomal locus occupied by the NORs was detected as a secondary constriction. NORs were localized on two pairs of metaphase chromosomes (Figure 2e). This organization was maintained during anaphase, when NORs moved with the chromosomes to the poles (Figure 2f–g). The newly forming nucleoli around the NORs were rebuilt in telophase. Finally, the two daughter nuclei entered interphase, and mitosis was completed (Figure 2h).

The nucleolar cycle during mitosis in root tip cells of *A. cepa* exposed to Pb was abnormal. The nucleoli in some cells exposed to low concentrations of Pb (1–10 μM) for 24–48 h did not disaggregate and retained their characteristic structures during metaphase (Figure 2i–j); they are referred to as persistent nucleoli. At a high concentration of Pb (100 μM Pb, 48 h), many silver-stained NOR particles were distributed on sticky chromosomes at metaphase (Figure 2k) and anaphase (Figure 2l).

### 2.3. Effects of Pb on Nucleoli

**Figure 3:**
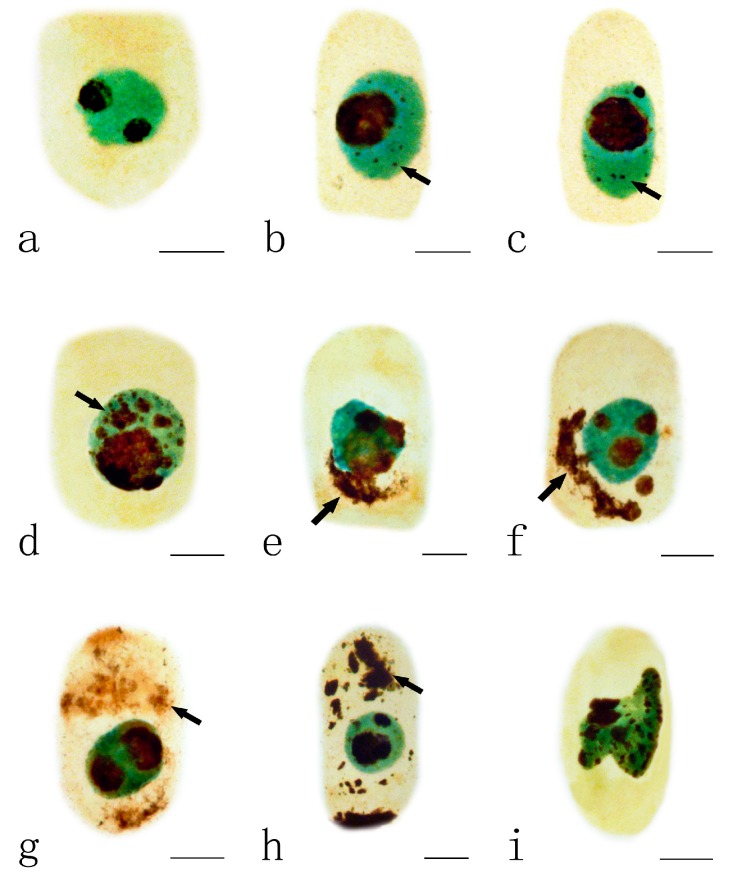
Effects of different concentrations of Pb on nucleoli in the root tip meristematic cells of *A. cepa*. (**a**) Control cell; (**b**,**c**) Silver-stained particles containing argyrophilic proteins in nucleus (10 μM Pb, 48 h); (**d**) Silver-stained particles accumulated in nucleus (100 μM Pb, 24 h); (**e**,**f**) Nucleolus particles extruded from the nucleus into the cytoplasm ((**e**) 10 μM Pb, 72 h; (**f**) 100 μM Pb, 48 h); (**g**,**h**) Nucleolar material in the cytoplasm ((**g**) 10 μM Pb, 72 h; (**h**) 100 μM Pb, 72 h); (**i**) Irregular nucleus (100 μM Pb, 48 h). Scale bars = 10 μm. Nucleoli and nucleolar material: dark brown; Nuclei: green; cytoplasm: yellow. Arrowheads show silver-stained particles.

Normally, the nucleus of *A. cepa* contains one or two dark-brown nucleoli (Figure 3a). The toxic effects of Pb on the nucleoli varied depending on the different concentrations and the treatment time. Swollen nucleoli with some tiny silver-stained particles were observed, first in the nucleus of the root tips exposed to 10 μM Pb for 48 h when compared to control cells (Figure 3b–c). With increasing Pb concentration (100 μM Pb, 24 h), more and more particles aggregated and nearly filled the nucleus (Figure 3d). On increasing the duration of treatment, these argyrophilic protein particles were extruded from the nucleus into the cytoplasm (Figure 3e–f) where they gradually accumulated to almost filling the cytoplasm (Figure 3g–h). Also, the shape of the nucleus was irregular in some cells exposed to 100 μM Pb for 48 h (Figure 3i).

### 2.4. Effects of Pb on Three Major Nucleoproteins

**Figure 4:**
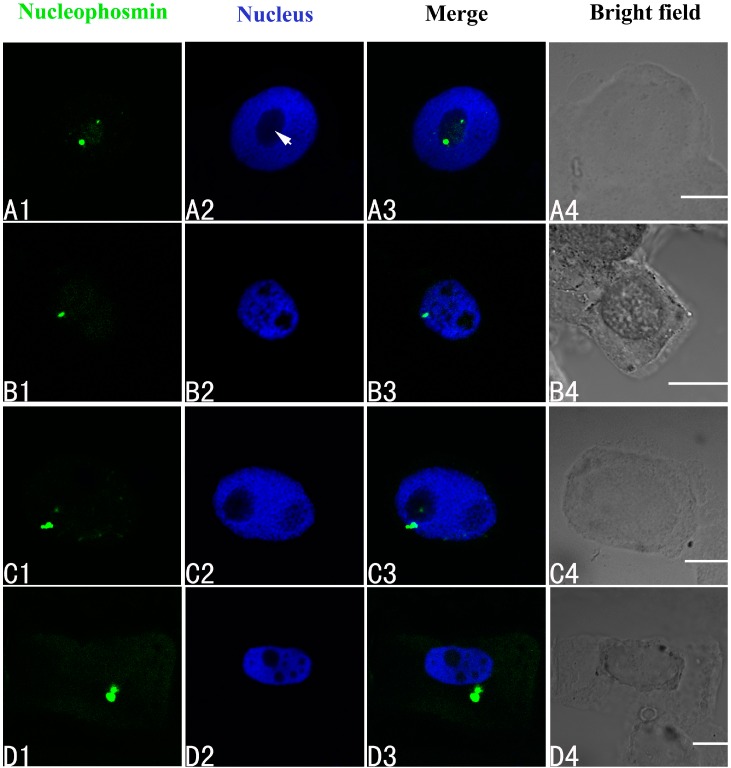
Simultaneous detection of nucleophosmin after incubation with primary anti-nucleophosmin antibody and secondary antibody conjugated with FITC (Fluorescein isothiocyanate) (green), and of DNA after incubation with DAPI (4',6-diamidino-2-phenylindole) (blue) in the same single optical section using confocal microscopy. (**A1**,**B1**,**C1**,**D1**) Nucleophosmin detection; (**A2**,**B2**,**C2**,**D2**) DNA detection; (**A3**,**B3**,**C3**,**D3**) Merged image of “nucleophosmin detection” and “DNA detection”; (**A4**,**B4**,**C4**,**D4**) bright field image. (**A1**–**A4**) Nucleophosmin mainly localized in the nucleolus of control cells; (**B1**–**B4**) The migration of nucleophosmin from the nucleolus to the nucleoplasm in cells treated with 100 μM Pb for 24 h; (**C1**–**C4**) The movement of nucleophosmin from the nucleoplasm into the cytoplasm in cells treated with 100 μM Pb for 48 h; and (**D1**–**D4**) Nucleophosmin in the cytoplasm of cells treated with 100 μM Pb for 72 h. Scale bars = 10 μm. Arrowhead shows nucleolus.

The effects of Pb on the three major nucleoproteins (nucleophosmin, nucleolin and fibrillarin) in the root tip meristematic cells of *A. cepa* were investigated using indirect immunofluorescence. First, it was clear that Pb was toxic. The biggest alteration in the localization of nucleophosmin was seen in the group treated with 100 μM Pb. Initially the nucleophosmin was found in the nucleolus (Figure 4A1–A4). Under Pb stress, the nucleophosmin migrated from the nucleolus to the nucleoplasm by 24 h (Figure 4B1–B4), and at 48 and 72 h, it had moved into the cytoplasm (Figure 4C–D).

**Figure 5:**
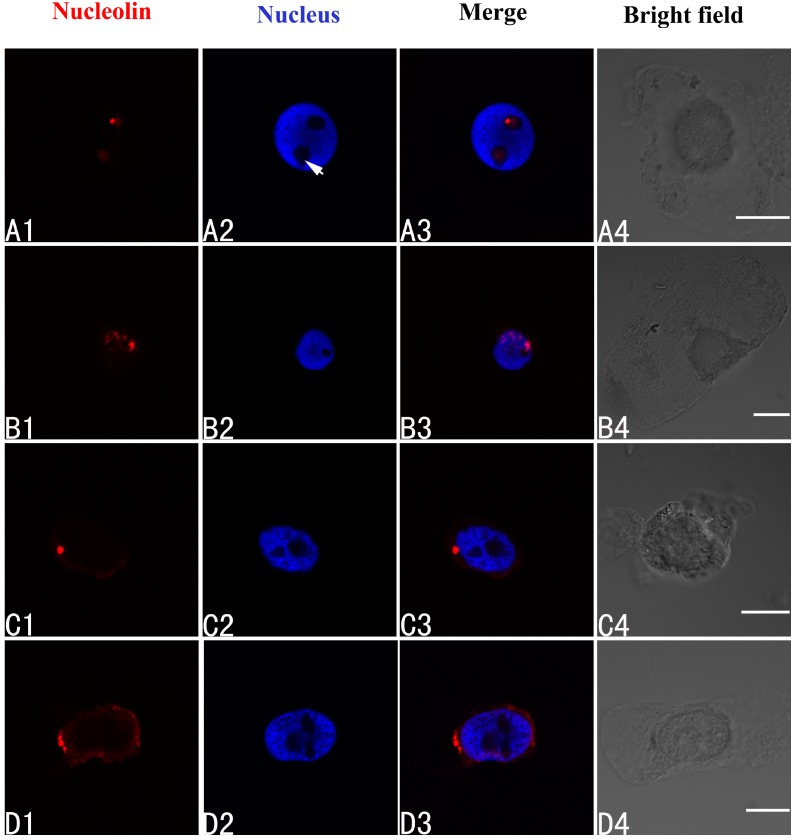
Simultaneous detection of nucleolin after incubation with primary anti-nucleolin antibody and secondary antibody conjugated with TRITC (Tetramethylrhodamine isothiocyanate) (red), and of DNA after incubation with DAPI (blue) in the same single optical section using confocal microscopy. (**A1**,**B1**,**C1**,**D1**) Nucleolin detection; (**A2**,**B2**,**C2**,**D2**) DNA detection; (**A3**,**B3**,**C3**,**D3**) Merged image of “nucleolin detection” and “DNA detection”; (**A4**,**B4**,**C4**,**D4**) bright field image. (**A1**–**A4**) Nucleolin in nucleolus of control cells; (**B1**–**B4**) The migration of nucleolin from the nucleolus to the nucleoplasm in cells treated with 100 μM Pb for 24 h; (**C1**–**C4**,**D1**–**D4**) Nucleolin in the cytoplasm of cells treated with 100 μM Pb for 48 h (**C1**–**C4**) and 72 h (**D1**–**D4**). Scale bars = 10 μm. Arrowhead shows nucleolus.

Nucleolin in control cells of *A. cepa* was localized in the nucleolus (Figure 5A1–A4). On treatment with 100 μM Pb for 24 h, nucleolin migrated from the nucleolus to the nucleoplasm (Figure 5B1–B4). Prolonging the treatment time led to nucleolin appearing in the cytoplasm (48 h) (Figure 5C1–C4) (48 h), and accumulating there (72 h) (Figure 5D1–D4).

**Figure 6:**
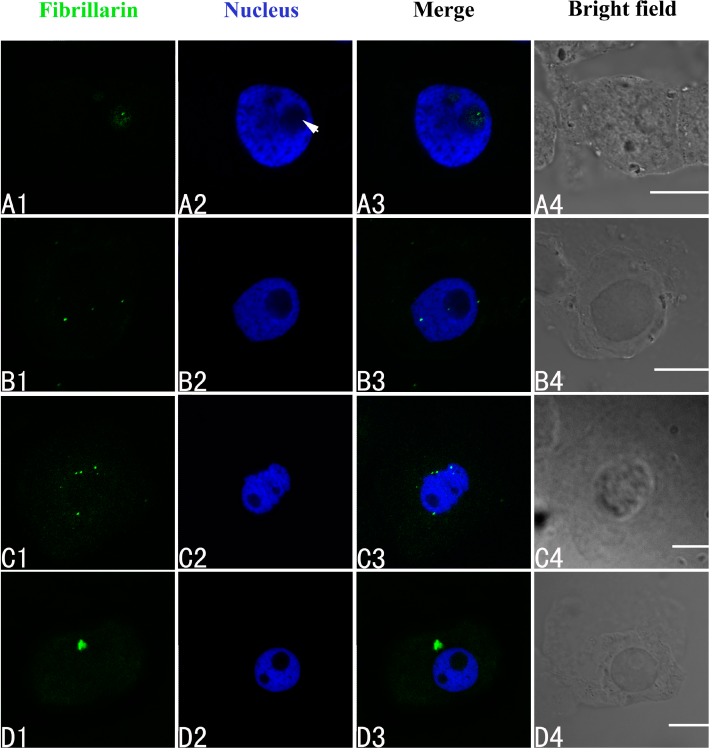
Simultaneous detection of fibrillarin after incubation with primary anti-fibrillarin antibody and secondary antibody conjugated with FITC (green), and of DNA after incubation with DAPI (blue) in the same single optical section using confocal microscopy. (**A1**,**B1**,**C1**,**D1**) Fibrillarin detection; (**A2**,**B2**,**C2**,**D2**) DNA detection; (**A3**,**B3**,**C3**,**D3**) Merged image of “fibrillarin detection” and “DNA detection”; (**A4**,**B4**,**C4**,**D4**) bright field image. (**A1**–**A4**) Fibrillarin in the nucleolus of control cells; (**B1**–**B4**) Migration of fibrillarin from the nucleolus to the nucleoplasm or the cytoplasm in the cells treated with 100 μM Pb for 24 h; (**C1**–**C4**) Fibrillarin in the cytoplasm of the treated cells at 48 h, and 72 h (**D1**–**D4**). Scale bars = 10 μm. Arrowhead shows nucleolus.

The intracellular distributions of fibrillarin were very similar to nucleophosmin and nucleolin using confocal laser scanning microscopy. Fibrillarin is normally present only in the nucleolus in untreated control cells of *A. cepa* (Figure 6A1–A4). In the group treated with 100 μM Pb for 24 h, disordered patterns of fibrillarin were observed, with transfer of fibrillarin from the nucleolus to the nucleoplasm or cytoplasm (Figure 6B1–B4). More fibrillarin was found in the cytoplasm by 48 h (Figure 6C1–C4), and a further increase by 72 h (Figure 6D1–D4).

### 2.5. Expression of Three Major Nucleoproteins in Relation to Pb Treatment

**Figure 7:**
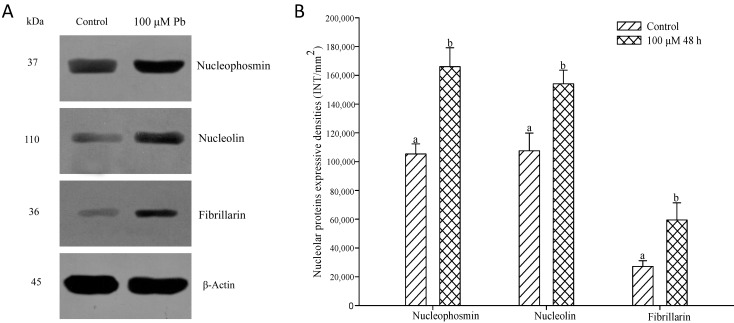
Effect of Pb (100 μM Pb, 48 h), on the expression of nucleophosmin, nucleolin and fibrillarin. Bands were detected by western blotting (**A**) and their intensities were measured by “Quantity One” software (Bio-Rad, Berkeley, CA, USA) (**B**). Bar indicates standard error (SE). *n* = 5. *p* < 0.05.

The levels of nucleophosmin, nucleolin and fibrillarin in root tip cells of *A. cepa* treated with 100 μM Pb were analyzed by western blotting (Figure 7). The expression of the proteins increased significantly after 48 h (*p* < 0.05) compared with the control, consistent with the results obtained by indirect immunofluorescent microscopy.

### 2.6. Discussion

Heavy metals such as Pb and Cd are ubiquitous environmental contaminants and exhibit widespread toxicity. The Intracellular Pb and Cd Detection Kit employs the Leadmium™ Green AM dye as a specific indicator of Pb or Cd in cells. Leadmium™ Green AM dye has been successfully used to detect Cd in plant roots [22,23,24], but, limited information on Pb in plants is available. Here we show that Pb enters the root tip meristematic cells of *A. cepa* after exposure to 100 μM Pb for 4 h. This supports our earlier findings [25] in which ultrastructural damage was observed in the root tip cells of *Allium sativum* exposed to 10^−4^ M Pb for 1–72 h. Thus the root tip meristematic cells of plants are a primary target of Pb toxicity.

The nucleolus is a highly dynamic subnuclear domain where rRNA synthesis, rRNA processing and assembly of ribosomal subunits take place [26]. It plays important roles in the regulation of many fundamental cellular processes, including cell cycle regulation, apoptosis, telomerase production, and the monitoring and response to cellular stress [27,28]. NORs contain a set of ribosomal genes and are associated with argyrophilic proteins [20,29]. The silver staining technique has been widely applied in cytological studies aimed at understanding the nucleolar cycle and its organization in both animals and plants [30]. Cellular activity in terms of transcriptional activity can be evaluated by measuring these argyrophilic NORs (AgNORs). In a typical cell nucleoli are formed around the ribosomal DNA (rDNA) repeats arranged around NORs on one or several chromosomes [31]. We found persistent nucleoli in some cells exposed to a low concentration of Pb, which is in a good agreement with the findings of Qin *et al.* [32], Zhang *et al.* [20] and Zou *et al.* [33] in which toxic effects of Al and Cd on NORs were investigated. As nucleolar persistence during mitotic metaphase and anaphase may cause increased biosynthetic activity and more protein production, thus rRNA synthesis continues in persistent nucleoli [34]. Vostrikova and Butorina [35] showed persistent nucleoli in some birch seedling cells during mitotic metaphase in response to anthropogenic stress (heavy metals, smoke and dust). Thus, it is possible that persistent nucleoli are a toxic response to the stress induced by Pb. With increasing concentration of Pb or prolonging the treatment time, the toxic characteristics of Pb resulted in many silver-stained particles being distributed on sticky chromosomes and in the cytoplasm during mitosis, due to the disintegration of the nucleoli. The effects of Pb on the nucleoli can seriously damage cells and even lead to cell death. Nucleolar segregation after Pb treatment in *Lupinus angustifolius* root meristematic cells has been reported [9]. Imazawa *et al.* [36] showed that nucleolar segregation occurred after the generation of DNA damage, which might indicate toxic effects, but not carcinogenic effects.

The initial site for a toxic metal to act is likely to be the cell membrane. Cations bound to the cell wall can be replaced efficiently by other cations with a stronger affinity for those binding sites. Calcium (Ca) and Pb have similar radii (Ca^2+^ 0.99 A; Pb^2+^ 1.25 A), so Ca can be replaced by Pb when plants absorb excessive Pb [37]. Pb toxicity has been closely linked to Ca metabolism and Ca-binding proteins [38]. Calmodulin (CaM) is Ca^2+^-binding protein that may play a role in Pb toxicity [39], as Pb will occupy the Ca^2+^ binding sites in CaM with higher relative affinity than Ca^2+^ [40]. Because of the result of cation competition, the level of free Ca in the cell will be very low and CaM will not activate Ca-ATPase [41], leading to failure in the regulation of Ca concentration and to disturbance of the physiological activities of CaM. Li and Sun [42] proposed that CaM might play a role in regulating and controlling RNA synthesis and nucleolar behaviour during interphase. Chen *et al.* [43] noted that, although there was more CaM in the cytoplasm, the nuclei, especially the nucleoli also contained CaM. These findings support the observations in the present investigation that silver-stained particles aggregated under Pb-stress and almost filled the nucleus before being released from the nucleolus into the cytoplasm as the Pb concentration and duration of treatment increased. Balcerzak *et al.* [9] showed by electron microscopy that the silver-stained particles in the nucleoli of *Lupinus angustifolius* L. root meristematic cells were released from the nucleus into the cytoplasm on exposure to Pb.

Our further experiments were designed to examine the nature of the silver-stained particles and what types of NPs were extruded from the nucleolus into the cytoplasm under Pb stress Using nucleolar mass spectrometry analyses, almost 700 NPs have been characterized. Some of these are required for ribosomal biogenesis [44]. Nucleophosmin, nucleolin and fibrillarin are major multifunctional nucleoproteins participating in rRNA processing and ribosome biogenesis [18]. Nucleophosmin, a 37-kDa phosphoprotein, plays a pivotal role in protecting cells from death in response to cell stress [45]. Various cellular activities have been attributed to it, including cell proliferation [46], nucleic acid binding, ribonuclease, molecular chaperone [47] and response to stress-stimuli [48]. Nucleolin is found in a diverse array of organisms ranging from yeast to plants to mammals. It is involved in rRNA processing, ribosome assembly, transcriptional repression, and transport of ribosomes to the cytoplasm [49], and may promote plant growth [50]. Nucleolin and nucleophosmin do not share any structural homology although they have numerous functions in common. The predominant location of these proteins in the nucleolus strongly suggests that they are involved in ribosome biogenesis. Fibrillarin is the most abundant protein in the fibrillar center of the nucleolus, with a key role in pre-rRNA processing during ribosomal biogenesis in eukaryotes [46]. Nucleophosmin and nucleolin are major silver-containing NOR proteins [51,52] and can be identified as black dots throughout the nucleolar area following silver staining. The results obtained in the present investigation by indirect immunofluorescence confirmed that the silver-stained particles extruded from the nucleolus into the cytoplasm in the root tip cells of *A. cepa* exposed to Pb contained nucleophosmin and nucleolin. Fibrillarin is distinguished from these two NPs by its lack of affinity for silver [53]. We found that Pb could induce an alteration in the distribution of fibrillarin in the root tips of *A. cepa* under Pb stress. Thus, we suggest that Pb, as is the case with other metals such as Al and Cd, has toxic effects on other types of NPs besides argyrophilic and acidic nucleoproteins [54,55].

Our results indicated that the three NPs were localized in nucleoli in the untreated root tips of *A. cepa*. On exposure to Pb they migrated from the nucleolus to the nucleoplasm or cytoplasm and were over-expressed. This increased expression may mirror an enhancement of nucleolar activity which is an important aspect of the cellular/nucleolar response to Pb stress. The abnormal cellular localization of the three NPs might explain the inhibition of rRNA synthesis induced by Pb. Thus, when cell growth and proliferation are depressed by Pb treatment, the nucleolar transcription is affected and its structure changed. Dundr [56] indicated that under stress conditions, many NPs change their locations, and their distribution inside and outside the nucleolus is disorderly. Treatment with actinomycin D causes dislocation of fibrillarin from the nucleolus to the nucleoplasm in tobacco cells [57]. HgCl_2_ dislocates fibrillarin from the nucleolus in human cells [58]. Inhibition of rRNA synthesis can affect the interaction between NPs and rRNA, inducing the relocation of the NPs [59]. Phosphatidylinositol 3-kinase (PI3K) is a crucial factor in the intracellular signal transduction pathway. It is distributed within the plant nucleus and nucleolus, being associated with active nuclear and nucleolar transcription sites in particular [60]. After the germinated seeds of *Triticum aestivum* L. were treated with wortmannin (a specific inhibitor of PI3K), PI3K was blocked, resulting in nucleophosmin moving from the nucleoli to the nucleoplasm or to the cytoplasm. This suggests that PI3K is not only involved in the structure of the nucleolus, but also in its function [61]. Dundr *et al.* [62] demonstrated that when RNA synthesis was inhibited, nucleophosmin relocated.

Using the Feulgen-light green method, Fiskesjö [63] observed that nucleolar material could be extruded from nucleus into the cytoplasm in *A. cepa* cells exposed to Al. The phenomenon was referred to as “Al-structure”. Following that finding, the toxic effects of heavy metals (Ni, Cd and Pb) on the nucleolus in the root tips of plants were investigated, indicating that these metals had a similar toxic effect on the nucleolus as Al [5,19,53,64,65,66]. More recent work using indirect immunofluorescence and western blotting indicated that nucleolar material migrated into the cytoplasm in the root tip cells of *A. cepa* exposed to Al [55] and of *Vicia faba* exposed to Cd [54]. The material contained nucleophosmin, nucleolin and fibrillarin. Our results suggest that the toxic effects of Pb on the nucleolus and nucleoproteins were the same as for Al and Cd, and, as such, represent early markers for the cellular changes induced by metals. They could also be used as signals for metal contamination and for evaluating the stress caused by metals.

## 3. Experimental Section

### 3.1. Culture Condition and Pb Treatment

Healthy onion bulbs of *A. cepa* of uniform size were chosen. Only bulbs without green leaves or any roots were selected. The dry scales of the bulbs were removed to expose the apices of the primordial root. The bulbs were propagated and grown in glass containers at 25 °C for 2 day, producing roots reaching about 1 cm length. Then the seedlings were grown in containers in solutions containing different concentrations of Pb (1, 10 and 100 μM Pb(NO_3_)_2_ at pH 5.5) for up to 72 h. The solutions were renewed every 24 h and aerated by a pump. The control seedlings were grown in distilled water alone. All treatments were done in triplicate.

### 3.2. Fluorescence Labeling

The intact roots of *A. cepa* seedlings treated with or without 100 μM Pb for 4, 24 and 48 h were stained using the Pb specific probe Leadmium™ Green AM dye (Molecular Probes, Invitrogen) according to the manufacturer’s instructions to visualize the Pb absorption and distribution. Roots were placed in 20 mM Na_2_-EDTA for 15 min at room temperature, then washed in ddH_2_O three times for 10 min each time. A stock solution of Pb-specific probe was made by adding 50 μL dimethyl sulfoxide to a vial of the dye. This was diluted 1:10 with 0.85% NaCl [24]. The roots were immersed in the diluted stock solution at 37 °C for 2 h in the dark, before washing with 0.85% NaCl three times. The roots were stored in the dark at 4 °C until visualization by confocal laser scanning microscopy using an exciter at 488 nm and a barrier filter at 590/50 nm.

### 3.3. Silver-Staining

Twenty root tips in each treatment group and control were cut and fixed in 95% ethanol and acetic acid (3:2) for 1 h and hydrolyzed in 1 M hydrochloric acid, 95% ethanol and 99.8% acetic acid (5:3:2) for 5 min at 60 °C. Then they were squashed in 45% acetic acid, dried and stained with silver nitrate or methylene blue two days later [19].

### 3.4. Immunofluorescence Staining

For the visualization of nucleophosmin, nucleolin and fibrillarin, meristematic zones in root tips of untreated *A. cepa* or treated with 100 μM Pb were cut and fixed with 4% (*w*/*v*) paraformaldehyde in phosphate-buffered saline (PBS, pH 7.0) for 2 h at room temperature, before washing with the PBS buffer three times for 10 min each time. The cell walls were digested with a mixture of 2.5% cellulase and 2.5% pectolase for 45 min at 37 °C, then washed three times in PBS. The cells were squashed on slides and extracted in freshly prepared 1% (*v*/*v*) Triton X-100 in PBS for 20 min. Following three washes in PBS, the cells were subsequently incubated with mouse primary antibodies specific for the three proteins for 1 h at 37 °C in a moist sealed chamber. After washing three times with PBS, the cells were incubated with secondary antibodies for 45 min at 37 °C in the dark. After repeated washing in PBS, nuclei were stained with 4',6-diamidino-2-phenylindole (DAPI, Sigma, San Francisco, CA, USA) at a final concentration of 1 μg/mL for 15 min at room temperature. The cells were mounted in an anti-fade solution after washing in PBS. The slides were stored in the dark at 4 °C until viewed. The antibodies were:

(1) Nucleophosmin: primary antibody: a mouse monoclonal antibody to nucleophosmin (Sigma, B0556) at dilution 1:100; secondary antibody: FITC-conjugated goat anti-mouse IgGs (Sigma, F9137) at dilution 1:50.

(2) Nucleolin: primary antibody: a mouse monoclonal antibody to nucleolin (Santa, Dallas, TX, USA, SC-8031) at dilution 1:100; secondary anti-body: TRITC-conjugated goat anti-mouse IgGs (Sigma, T5393) at dilution 1:50.

(3) Fibrillarin: primary antibody: a mouse monoclonal antibody to fibrillarin (Santa, SC-166001) at dilution 1:100; secondary antibody: FITC-conjugated goat anti-mouse IgGs (Sigma, F9137) at dilution 1:50.

The specimens were examined with a confocal laser scanning microscope (Nikon ECLIPSE 90i, Nikon Inc., Melville, NY, USA). An exciter at 408 nm and a barrier filter at 515/30 nm, an exciter at 488 nm and a barrier filter at 590/50 nm and an exciter at 543 nm and a barrier filter at 650 nm were used for DAPI, FITC and TRITC staining, respectively. Images were recorded using software EZ-C1 3.80 (Nikon Inc., Melville, NY, USA) according to the manufacturer’s instructions.

### 3.5. Western Blotting

Control root tips and those treated with 100 μM Pb for 48 h were washed three times in ddH_2_O and homogenized with liquid nitrogen in mortars. The homogenates were dissolved in chilled extraction buffer (50 mM Tris-HCl (pH 7.8), 10 mM MgCl_2_, 20 mM β-mercaptoethanol, 1.0 mM EDTA, 8% glycerol) with the addition of protease inhibitor cocktail set VI (Merck, Darmspadt, Germany, 539133). After stirring for 1 min at room temperature, the homogenates were kept on ice for 30 min, and then centrifuged at 10,000 rpm at 4 °C for 10 min. Three parts of the supernatant mixed with one part of 4× laemmli buffer (62.5 mM Tris-HCl (pH 6.8), 5% β-mercaptoethanol, 2% SDS (sodium dodecyl sulfate), 10% glycerol, 0.001% bromophenol blue) was boiled at 100 °C for 7 min. Equal amounts of protein were loaded on to each well and subjected to 12% SDS-PAGE (polyacrylamide gel electrophoresis). The separated proteins were transferred to 0.45 μm PVDF (polyvinylidene fluoride) transfer membrane (Millipore, Darmspadt, Germany, IPVH00010) using a semi-dry transfer apparatus. The membrane was blocked for 2 h with 5% (*w*/*v*) non-fat milk in Tris-buffered saline Tween (TBST) at room temperature with gentle shaking and then incubated with the primary antibodies described above, diluted in TBST buffer (nucleophosmin, 1:4000; nucleolin, 1:1000; fibrillarin, 1:750) at room temperature for 2.5 h. Anti-β-actin monoclonal antibody (Abmart, Shanghai, China, P30002) was used for the internal control. The membranes were washed in TBST buffer two times and TBS (Tris-buffered saline) buffer once, for 10 min on each occasion. The appropriate HRP (Horseradish peroxidase)-conjugated secondary antibody, also diluted in TBST buffer (1:7000), was added for 1.5 h at room temperature. Another three washes removed excess antibody. Finally, specific bands were detected using the ECL (Electrochemiluminescence) reagent (Millipore, WBKL S0100) and exposed to an X-ray film. The intensities of the bands in the film were quantitated by software ‘‘Quantity One’’ (Bio-Rad).

### 3.6. Statistical Analysis

Analysis of variance of data was performed using SPSS 15.0 version (SPSS, Chicago, IL, USA) for Windows software. For statistical analysis, the independent-samples *t*-test was used to determine the significance at *p* < 0.05.

## 4. Conclusions

Results from the present investigation demonstrated that the root tip meristematic cells of *A. cepa* plants were primary targets of Pb toxicity. Under Pb stress, Pb could disturb the nucleolar cycle with the extrusion of silver-stained materials containing argyrophilic proteins from the nucleolus into the cytoplasm. Indirect immunofluorescence detected nucleolar material containing nucleophosmin, nucleolin and fibrillarin and their movement into the cytoplasm following Pb stress. Western blotting revealed higher expression of these three major nucleoproteins in Pb-treated roots, which was consistent with the results obtained by indirect immunofluorescence.
